# HERV-E ƛ 4-1 activation in peripheral blood mononuclear cells of the recurrent depression patients under the influence of human recombinant IL-1β

**DOI:** 10.1192/j.eurpsy.2024.411

**Published:** 2024-08-27

**Authors:** E. Markova, I. Goldina, B. Goldin

**Affiliations:** ^1^Neuroimmunology Lab, State Research Institute of Fundamental and Clinical Immunology; ^2^Novosibirsk State Medical University, Novosibirsk, Russian Federation

## Abstract

**Introduction:**

Mental disorders represent complex phenotypes and are the leading causes of global disease burden. Human endogenous retroviruses (HERVs) are ancient retroviral DNA sequences established into germline. Their tight regulation is mainly achieved by epigenetic mechanisms, which can be altered by environmental factors - viral infections, inflammation, leading to HERV activation. The aberrant expression of HERVs associates with neurological diseases and mood disorders. We showed earlier that HERV-E ƛ 4-1 activation is associated with the recurrent depression stage of exacerbation and are accompanied by a pronounced increase in the proinflammatory activity of the peripheral blood mononuclear cells (PBMC).

**Objectives:**

The purpose of the study was to evaluate the activity of HERV-E ƛ 4-1 on PBMCs of patients with recurrent depression in remission, including under the influence of recombinant human IL-1β.

**Methods:**

The study included 30 patients with an established diagnosis of recurrent depression (F 33.0) aged 26–45 years. PBMC were isolated using the Ficoll density gradient method and further cultured in the presence or absence of 1 mkg/ml of recombinant human IL-1β for 24 hours. HERV-E λ 4 – 1 env gene expression was determined by the PCR. Cells proliferative activity was determined by H^3^-thymidine incorporation. Cytokines content in culture supernatants was assessed by ELISA.

**Results:**

It was shown that in all samples of PBMC cultured without IL-1β the HERV-E λ 4 – 1 env expression was not determined. After the PBMC cocultivation with recombinant human IL-1β, HERV-E λ 4 – 1 env gene expression was determined in 86,7% of cases. The HERV-E ƛ 4-1 activation in PBMC after IL-1β influence was accompanied by increased cells proliferative activity and production of IL-1β, IL-6.

**Conclusions:**

Our data indicate that the HERV-E λ 4 – 1 env expression in PBMC of recurrent depression patients in the stage of remission induced by the influence of proinflammatory cytokines, such as IL-1β. This mechanism may be one of the possible regulators of HERV-E λ 4 – 1 activation in recurrent depression.

**Disclosure of Interest:**

None Declared

